# Single-cell RNA sequencing identifies uterine stromal cells as a previously unrecognized target of an alphacoronavirus underlying reproductive disorders

**DOI:** 10.1128/jvi.00237-26

**Published:** 2026-07-22

**Authors:** Xianhui Liu, Lin Wang, Yilong Liu, Moukang Xiong, Mengpo Zhao, Leyi Zhang, Yanling Liu, Pengshuai Liang, Hanqin Shen, Linjun Hong, Zheng Xu, Changxu Song

**Affiliations:** 1 State Key Laboratory of Swine and Poultry Breeding Industry, National Engineering Research Center for Breeding Swine Industry, College of Animal Science, South China Agricultural University 12526, Guangzhou, China; 2 College of Veterinary Medicine, South China Agricultural University 12526, Guangzhou, China; 3 Wen’s Foodstuff Group Co. Ltd, Guangdong Enterprise Key Laboratory for Animal Health and Environmental Control, Yunfu, Guangdong Province, China; Loyola University Chicago - Health Sciences Campus, Maywood, Illinois, USA

**Keywords:** coronavirus, porcine epidemic diarrhea coronavirus, reproductive disorders, stromal cells, pathogenesis

## Abstract

Porcine epidemic diarrhea virus (PEDV), an alphacoronavirus, is primarily recognized as an enteric pathogen causing severe gastrointestinal disease in newborn piglets. PEDV has been endemic in most parts of the world, leading to high mortality in neonatal piglets and causing massive economic losses to the pig industry. However, reproductive disorders have been repeatedly observed in sows following PEDV outbreaks, and the underlying mechanisms remain poorly understood. Here, we provide evidence that PEDV can infect the porcine uterus *in vivo* and exhibits a pronounced cellular tropism toward uterine stromal cells. Using single-cell RNA sequencing, immunofluorescence analyses, and primary cell infection models, we show that PEDV-derived viral transcripts and proteins are preferentially detected in stromal cells, whereas epithelial and other uterine cell populations display minimal viral signals. PEDV efficiently replicates in primary porcine uterine endometrial cells in a trypsin-independent manner, indicating tissue-specific differences in viral entry requirements. Transcriptomic profiling reveals that PEDV infection is associated with extensive inflammatory activation, disruption of extracellular matrix organization, and altered intercellular communication. Notably, stromal cells emerge as a central signaling hub within the infected primary uterine endometrial cell culture system and likely within the uterine microenvironment as well. Correlation analyses further suggest that PEDV infection is linked to stromal cell-restricted expression patterns of candidate host factors rather than canonical enteric coronavirus receptors. Together, these findings redefine the cellular landscape of PEDV infection beyond the intestine and provide a mechanistic framework linking PEDV infection of uterine stromal cells to PEDV-associated reproductive disorders.

**IMPORTANCE:**

This study identifies uterine stromal cells as a novel target of porcine epidemic diarrhea virus (PEDV), breaking the long-held notion that PEDV is strictly enteric and expanding its known tissue tropism beyond the intestine. Leveraging single-cell RNA sequencing and multi-layered functional validation, it uncovers that PEDV replicates efficiently in uterine stromal cells via a trypsin-independent mechanism—distinct from its intestinal infection mode. This infection triggers inflammatory activation, extracellular matrix disruption, and altered intercellular communication, which are potential key contributors of PEDV-associated reproductive disorders plaguing the pig industry. These findings redefine alphacoronavirus pathogenesis and provide a critical mechanistic framework for developing targeted interventions. Additionally, this work highlights the value of single-cell approaches in unraveling extraintestinal viral infection, advancing both coronavirus biology research and veterinary disease control.

## INTRODUCTION

Porcine epidemic diarrhea virus (PEDV) belongs to the Alphacoronavirus genus in the Coronaviridae family (1, 2). PEDV primarily causes acute enteric disease in neonatal piglets, leading to severe diarrhea with high mortality (3, 4). It has been endemic in most parts of the world since its emergence in the 1970s, causing massive economic losses to the pig industry (3–5). Extensive efforts have focused on understanding PEDV replication, transmission, and pathogenesis within the intestinal tract (6, 7). However, accumulating field observations indicate that PEDV outbreaks are frequently accompanied by reproductive disorders in gilts and sows, including decreased conception rates, abortion, and stillbirth (8, 9). Despite these recurring clinical observations, the biological basis underlying PEDV-associated reproductive dysfunction remains poorly understood.

Coronaviruses are increasingly recognized as pathogens capable of infecting multiple tissues beyond their primary target organs (10, 11). Extraintestinal and extrapulmonary manifestations have been documented for several coronaviruses (10–12), highlighting the importance of tissue-specific viral tropism in shaping disease outcomes. In contrast, PEDV has long been considered a strictly enteric virus (6, 7), and whether it directly infects reproductive tissues or specific uterine cell populations has not been systematically examined. In addition, the receptor and entry mechanisms of PEDV are not clear, which also increases the difficulty in studying the pathogenic mechanism of PEDV (6, 7).

The uterus is a highly specialized organ composed of heterogeneous cell populations that coordinate immune regulation, extracellular matrix remodeling, and vascular dynamics to support normal reproductive function (13, 14). Among these, uterine stromal cells play a central role in maintaining endometrial integrity and orchestrating tissue responses during implantation and pregnancy (14, 15). Disruption of stromal cell function is known to impair uterine homeostasis and has been implicated in various reproductive disorders (14, 15).

Single-cell RNA sequencing (scRNA-seq) provides a powerful approach to dissect tissue complexity and identify virus–host interactions at cellular resolution (16–19). By enabling simultaneous detection of viral transcripts and host gene expression across thousands of individual cells, scRNA-seq offers a unique opportunity to define tissue-specific viral tropism and infection-associated cellular responses that are otherwise obscured in bulk analyses (16–18, 20).

In this study, we applied scRNA-seq in combination with *in vivo* infection analyses, immunofluorescence validation, and primary uterine cell infection models to investigate PEDV infection in the porcine uterus. We identify uterine stromal cells as a previously unrecognized cellular target of PEDV and show that infection of these cells is associated with inflammatory activation, extracellular matrix remodeling. Collectively, our results reveal a cellular and tissue tropism of PEDV extending beyond the intestinal tract and establish a mechanistic basis for elucidating PEDV-associated reproductive disorders.

## RESULTS

### PEDV was identified from the uterine organs of sows infected with PEDV

**Fig 1:**
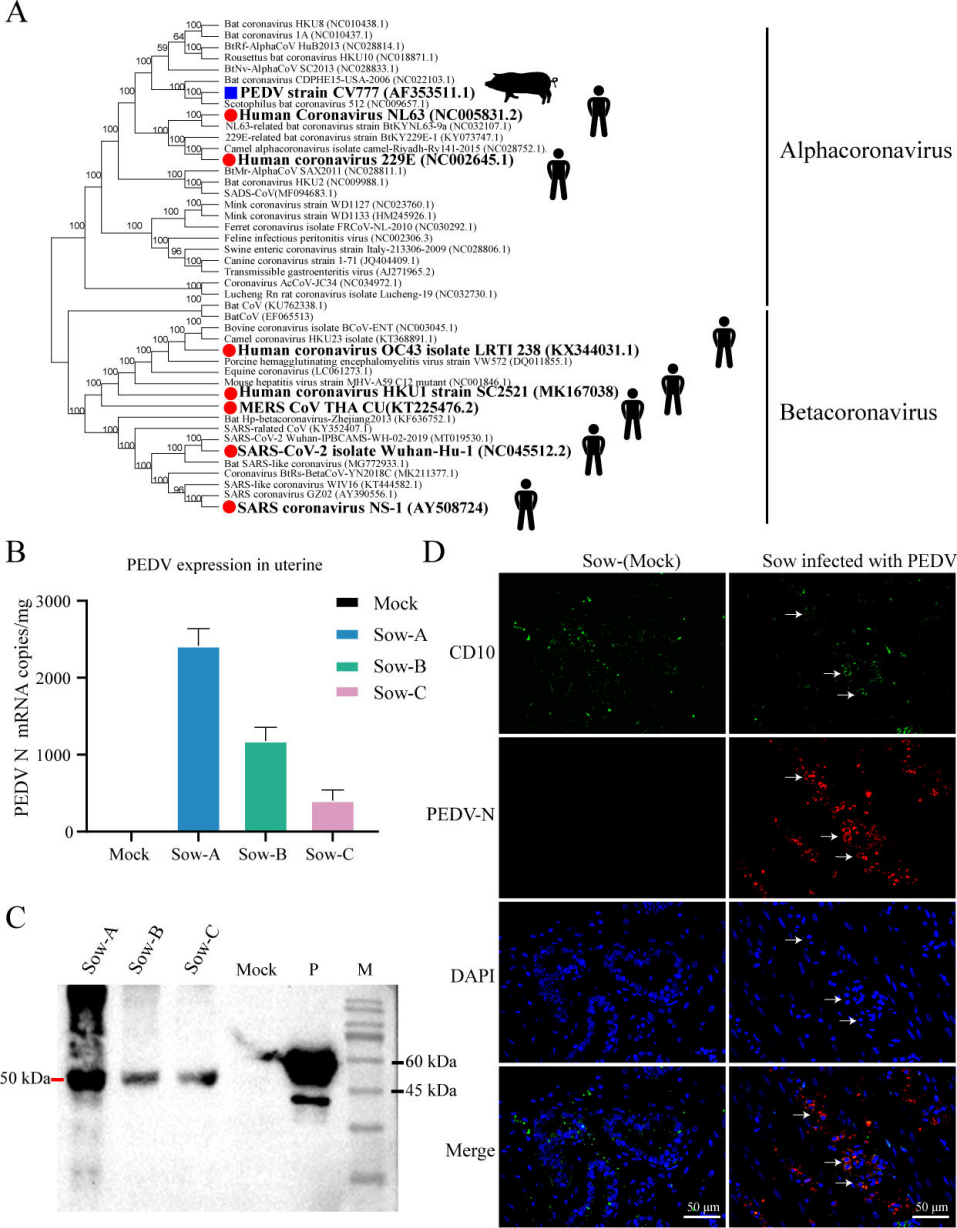
PEDV was identified from the uterine organs of sows infected with PEDV. (**A**) Bayesian phylogenetic tree of the full-length genome of PEDV and related coronaviruses, the blue square represents the PEDV strain, while the red circle represents human coronavirus strains. (**B**) PEDV-N mRNA copies were detected in the uterine tissue of sow A, sow B, and sow C infected with PEDV. (**C**) PEDV nucleocapsid protein was detected by western blot in uterine tissue of sows infected with PEDV. The Sow-A, Sow-B, and Sow-C indicate uterine tissues of sow A, sow B, and sow C infected with PEDV, the Mock indicates negative sows not infected with PEDV, and P represents a positive control. (**D**) PEDV infects uterine tissue of sows with CD10 as a marker. CD10 (green), PEDV-N (red), DAPI (blue).

Phylogenetic analysis of the full-length genome demonstrated considerable genetic diversity among bat coronaviruses and revealed pronounced coevolutionary relationships with their hosts (Fig. 1A). Furthermore, it showed that PEDV, which belongs to the alphacoronavirus subfamily, is most closely related to a bat coronavirus derived from *Scotophilus* bats. In response to the observed rise in reproductive disorders in PEDV-infected sows, we conducted experimental studies to address this question, and three naturally PEDV-infected sows were selected for analysis. These sows infected with PEDV had farrowed one week prior, and their piglets exhibited severe diarrhea and tested positive for PEDV. When the sows’ feces had a high virus titer, the virus titer in the uterus was detected. As shown in Fig. 1B, high PEDV-N mRNA copies were detected in the uterine tissue of sow A, sow B, and sow C infected with PEDV. Besides, Western blotting indicated that the PEDV-N protein was detected in uterine tissue of sows infected with PEDV (Fig. 1C). Moreover, IFA results showed that there were PEDV virus particles in the sow’s uterus (Fig. 1D), the colocalization of PEDV particles and CD10 further indicated that PEDV infected stromal cells in uterine tissue of sows. These results further reveal uterine stromal cells in sows infected with PEDV *in vivo*.

### PEDV efficiently replicates in primary porcine endometrial cells

**Fig 2:**
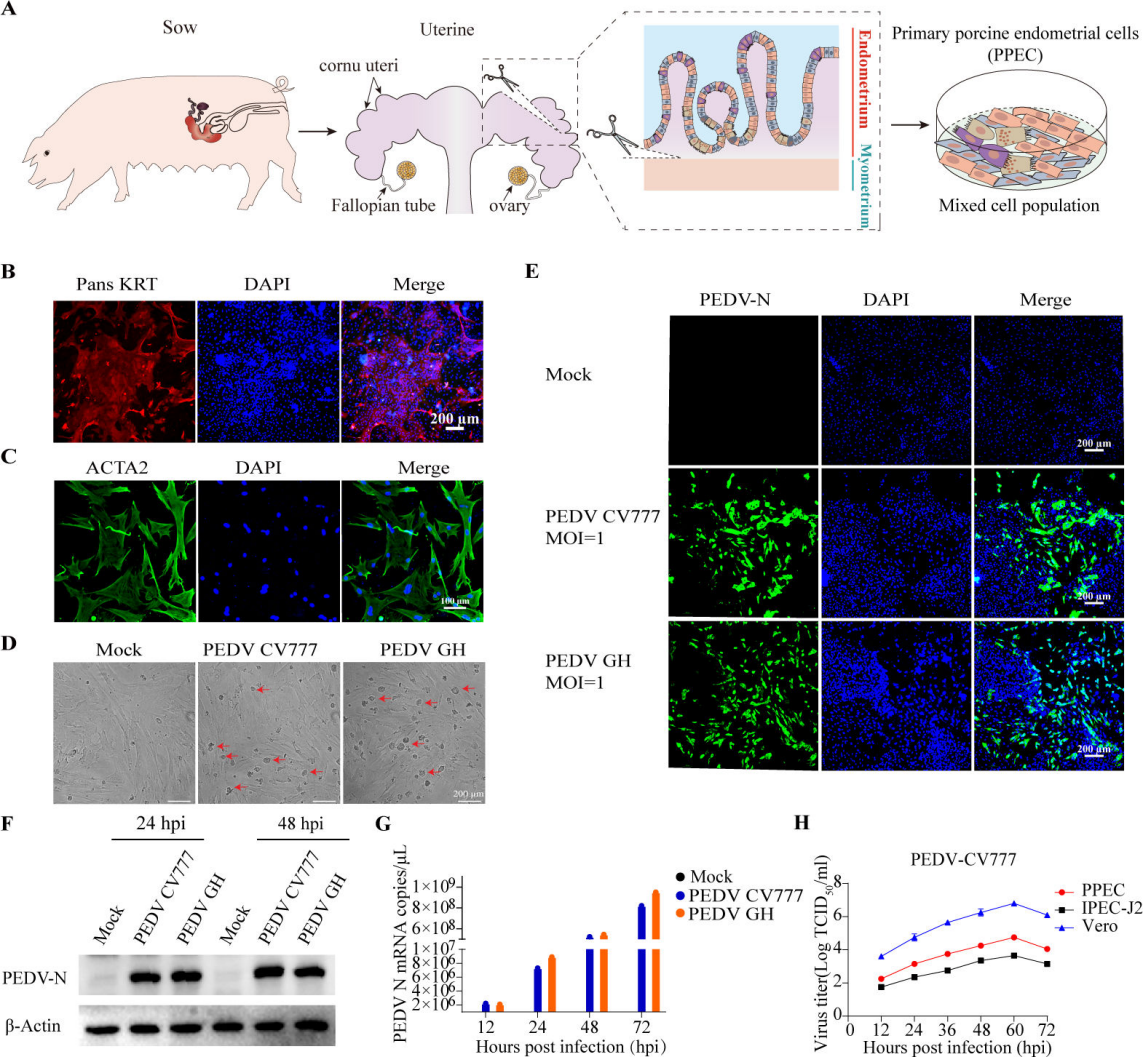
PEDV efficiently replicates in primary porcine endometrial cells (PPEC). (**A**) The flow chart of isolation and culture of primary porcine endometrial cells (PPEC). (**B**) Identification and analysis of the PPEC by IFA. Images of PPEC are representative of cells marked with porcine endometrial cells, Pans-KRT (red). (**C**) The epithelial α-smooth muscle actin rabbit monoclonal antibody (ACTA2) (green) positive phenotype is evident in PPEC. (**D**) PPEC infected with PEDV CV777 and PEDV GH at MOI = 1 at 48 h post infection. The red arrow represents the dead cells. (**E**) IFA indicates PEDV-N protein positive signals in PPEC infected with either CV777 strain and GH strain at MOI = 1 without trypsin solution, respectively. (**F**) Western blot indicates that the PEDV nucleocapsid protein in PPEC infected with PEDV. (**G**) PEDV-N mRNA copies of PPEC infected with PEDV were analyzed by RT-PCR. (**H**) The TCID_50_ of PEDV CV777 strain in Vero, IPEC-J2, and PPEC.

Subsequently, we aimed to further investigate the infection characteristics of PEDV in the uterine target organ. We attempted to isolate and culture primary porcine endometrial cells (PPEC). In the uterine endometrial fragments, upon mechanical separation, we observed the presence of well-preserved endometrium along with residues of the myometrium smooth muscle (Fig. 2A). Epithelial cells and glandular fragments were isolated from uterine tissues of sows using Collagenase Type I digestion (Fig. 2A). Under a defined culture system utilizing Matrigel and WNT-activation conditions, these cells failed to self-organize or form organoid-like structures capable of further expansion (data not shown). This could be due to the presence of certain mesenchymal cells mixed within the epithelial cells, preventing the formation of organ-like structures. After 3 days of culture, the PPEC displayed an adaptation phase. The positive expression of ACTA2 (α-Smooth Muscle Actin) and pan-cytokeratin (Pans KRT) (14, 21) was observed in the PPEC (Fig. 2B and C).

To demonstrate that PEDV is capable of infecting PPEC, the cell infected with PEDV CV777, an attenuated virus that belongs to subtype G1, and field PEDV GH strain that belongs to subtype G2 (S1. Fig). At a multiplicity of infection (MOI) of 1, PPEC inoculated with the two PEDV strains exhibited mild cytopathic effects (CPE) and a small number of floating dead cells at 48 h post-infection (hpi) (Fig. 2D). No typical syncytium formation was observed in PEDV-infected PPECs. However, compared with uninfected control cells, PEDV-infected cells showed an increased tendency to become rounded, and the number of round, floating cells in the supernatant was also elevated (Fig. 2D). After PPEC was infected with PEDV CV777 or GH without trypsin solution, a strong PEDV-N positive signal was observed (Fig. 2E). The addition of trypsin to the culture medium did not significantly increase the viral titer during infection (S2 Fig). Besides, the Western blotting assay indicated that the PEDV-N protein was detected in PPEC infected with PEDV (Fig. 2F). Moreover, PEDV-N protein mRNA copies of PPEC infected with PEDV illustrated PEDV infect PPEC effectively (Fig. 2G). Vero cells are commonly used cell lines for isolation and passage of PEDV. Under natural conditions, PEDV mainly infects porcine intestinal epithelial cells, which are the target cells of PEDV infection *in vivo*. Notably, the PEDV CV777 showed a peak virus titer in both Vero cells and PPEC but replicated weakly in IPEC-J2 cells (Fig. 2H). These results further confirm that PEDV can effectively infect and replicate in PPEC.

### Single-cell transcriptomic analysis reveals uterine stromal cells as the primary target of PEDV infection

**Fig 3:**
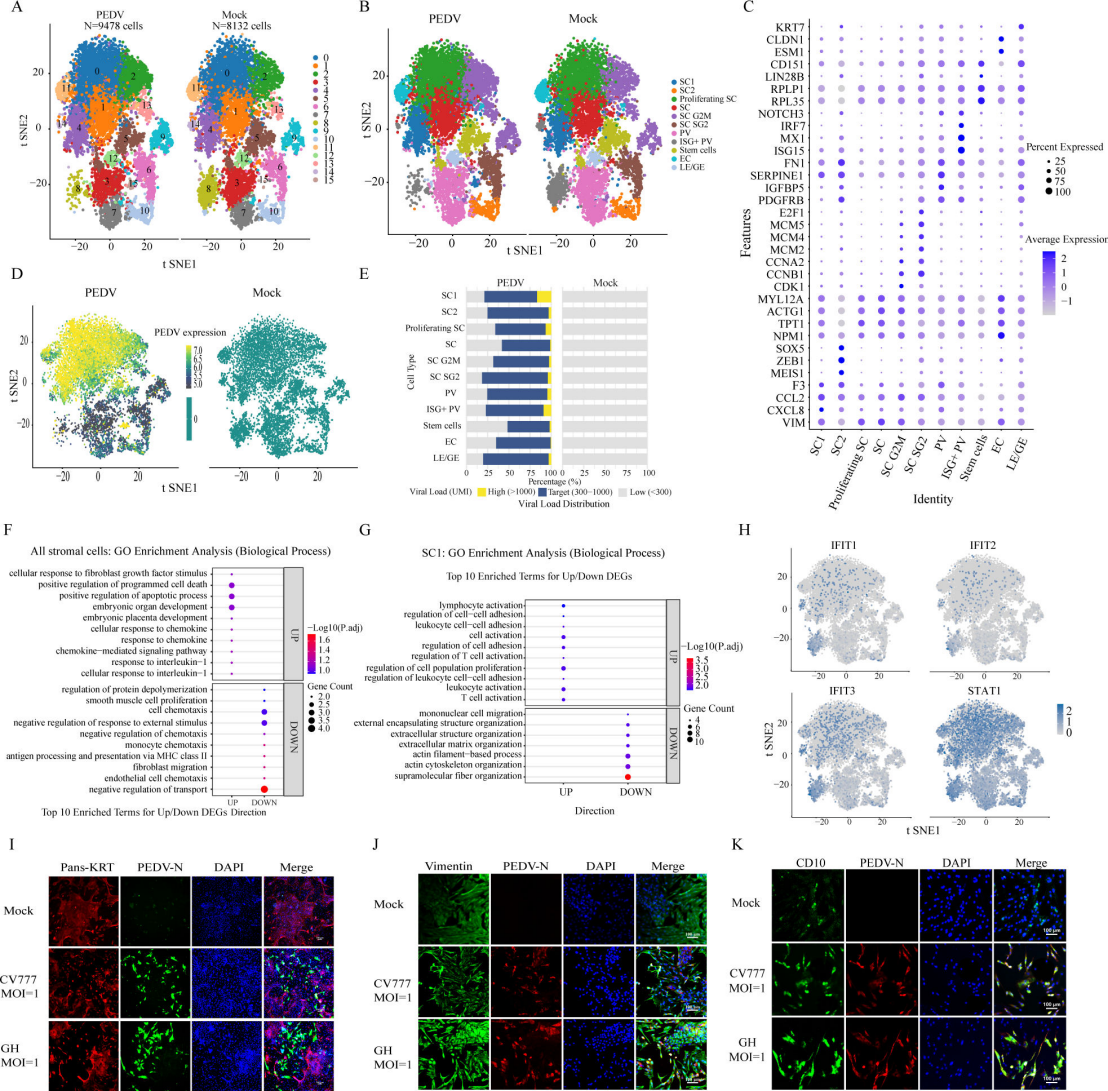
Single-cell transcriptomic analysis reveals uterine stromal cells as the primary target of PEDV infection. (**A**) Uniform manifold approximation and projection (UMAP) visualization of single cells isolated from PPEC. (**B**) Uniform manifold approximation and projection (UMAP) visualization of single cells isolated from PPEC, colored according to annotated cell types, including stromal cells, epithelial cells, endothelial cells, vascular endothelial cells, and stem cells. (**C**) Dot plot showing the mean expression of marker genes used to classify cell clusters. The color intensity indicates gene expression levels, whereas dot size represents the fraction of cells expressing the gene within each cell type. (**D**) Feature plot showing the distribution of PEDV-derived viral transcripts across uterine cell populations. PEDV RNA signals are predominantly localized to stromal cells. (**E**) Proportion of PEDV-infected cells within each uterine cell type, indicating selective enrichment of viral infection in stromal cells. Proportion of PEDV-positive cells across different cell types. (**F**) The bubble size indicates the absolute gene counts of all stromal cells enriched in a GO term, and the color bar indicates the minus logarithm of *q* values. (**G**) The bubble size indicates the absolute gene counts of stromal cells-1 (SC-1) enriched in a GO term, and the color bar indicates the minus logarithm of *q* values. (**H**) UMAP visualization showing the expression levels of IFIT1, IFIT2, IFIT3, and STAT1 in antiviral response genes from PEDV-infected (red) groups based on single-cell RNA-seq data. (**I**) PEDV does not infect PPEC with keratin as a marker. The red indicates keratin, and the green indicates PEDV N expression. (**J**) PEDV infects PPEC with vimentin as a marker. The green indicates vimentin, and the red indicates PEDV N expression. (**K**) PEDV infects PPEC with CD10 as a marker. The green indicates CD10, and the red indicates PEDV N expression.

To determine the cellular tropism of porcine epidemic diarrhea virus (PEDV) in the porcine uterus, single-cell RNA sequencing (scRNA-seq) was performed on PPEC collected after PEDV infection. Isolated primary porcine uterine endometrial cells are often not composed of a single cell type but rather a mixed cell population (Fig. 3A). Uterine epithelium is a complex tissue, including a variety of different types of cells, mainly composed of epithelial cells and stromal cells and other specialized types of cells (Fig. 3B). After stringent quality control, unsupervised clustering analysis identified multiple major uterine cell populations, including stromal cells, epithelial cells, endothelial cells, vascular endothelial cells, and stem cells, which were annotated based on canonical marker gene expression (Fig. 3A through C). Mapping of PEDV-derived viral transcripts onto the cellular atlas revealed a highly restricted distribution pattern. Notably, PEDV RNA signals were predominantly detected in uterine stromal cells, whereas epithelial cells and other cell types exhibited minimal or undetectable viral expression (Fig. 3D). Quantitative analysis further demonstrated that both the proportion of PEDV-positive cells and the viral transcript abundance were significantly higher in stromal cells compared with other uterine cell populations (Fig. 3E). It is noteworthy that although all stromal cells (including SC1, SC2, proliferating SC, SC, SCG2M, and SCSG2) remain the primary target of PEDV infection (Table S2 and S3), PEDV genes have also been detected at varying levels in other cell types, including perivascular cells with ISG highly expressed, luminal epithelial cell (LE), glandular epithelial cell (GE), etc. (Fig. 3D and E). It also indicated that all stromal cells (including SC1, SC2, proliferating SC, SC, SCG2M, and SCSG2) accounted for the largest proportion of all PPEC cells before and after PEDV infection, being 59.875% and 76.926%, respectively (Table S2 and S3). Based on the gene expression profiles of differentiated cells (20), the stromal cells were classified more precisely. The results showed that PEDV had the highest expression level in stromal cell type 1 (SC1) (Fig. 3D and E). Consistent with viral tropism, all stromal cells displayed distinct transcriptional alterations, including the upregulation of genes associated with antiviral responses and inflammatory signaling pathways (Fig. 3F), and stromal cells-1 (SC-1) also displayed distinct transcriptional alterations (Fig. 3G). In contrast, results showed IFIT1, IFIT2, IFIT3, and STAT1 transcriptional changes in PPEC infected with PEDV, supporting the notion that stromal cells represent the primary target cell type for PEDV infection in the porcine uterus (Fig. 3H).

In addition, it has been observed that not all isolated cells are infected by PEDV (Fig. 2E). Further identification is needed to determine which specific cells are susceptible to PEDV infection, and the results also reveal that PEDV does not infect epithelial cells because PEDV viral particles do not co-localize with the epithelial cells marked by pan-keratin (Fig. 3I). Subsequently, we have further confirmed that PEDV infects stromal cells, as PEDV viral particles co-localize with vimentin (22), a marker for stromal cells (Fig. 3J). Further results also showed that PEDV also co-localizes with another stromal cell marker, CD10 (15) (Fig. 1D and 3K). Collectively, these data demonstrate that PEDV exhibits a strong tropism toward uterine stromal cells in the porcine uterus.

### Cell–cell communication network analysis reveals uterine stromal cells as a central signaling hub following PEDV infection

**Fig 4:**
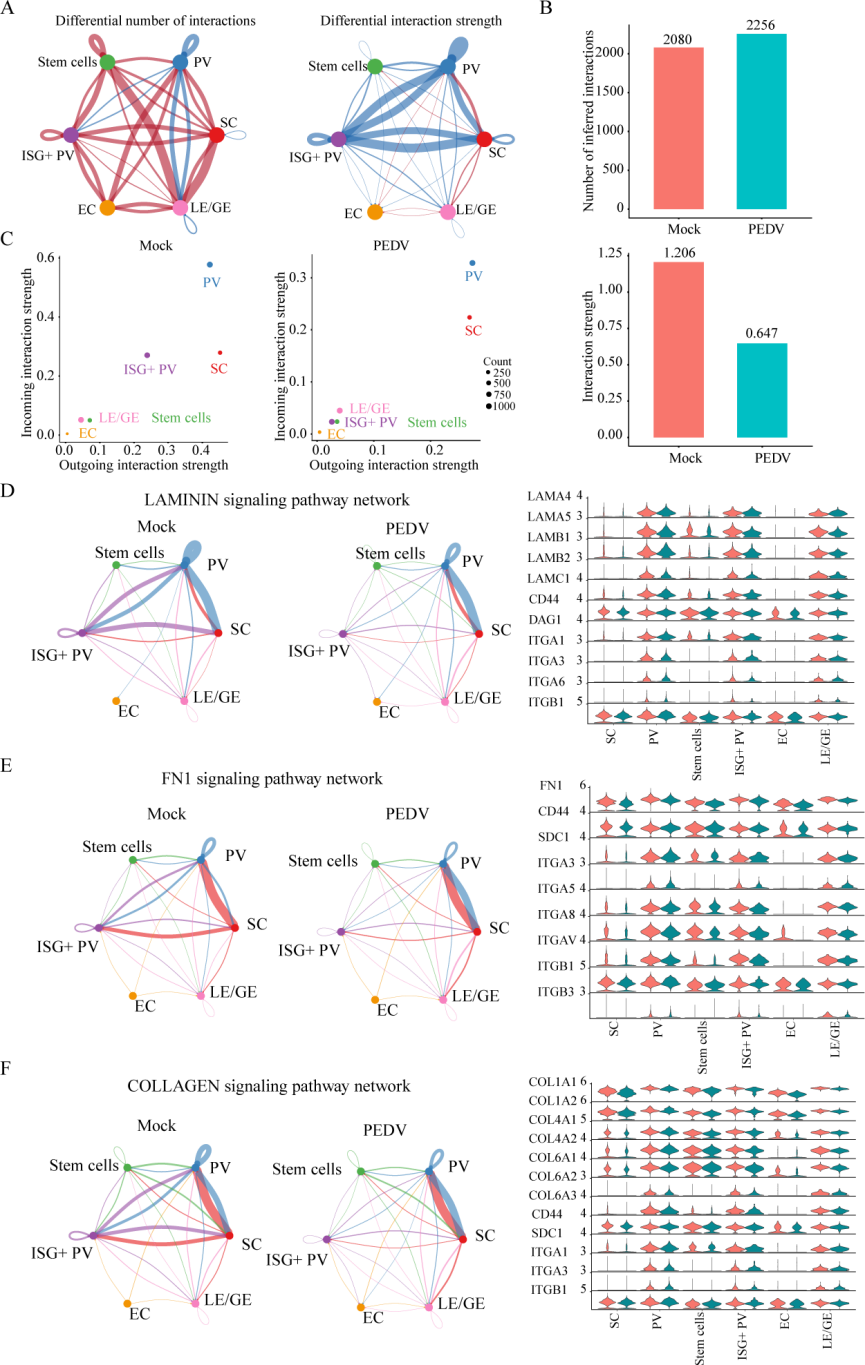
Cell–cell communication network analysis reveals uterine stromal cells as a central signaling hub following PEDV infection. (**A**) Global overview of intercellular communication networks among major uterine cell populations inferred from ligand-receptor pair analysis. Line thickness represents interaction strength, and arrow direction indicates signal flow from sender to receiver cells. (**B**) Quantification of outgoing and incoming signaling strength for each uterine cell type, highlighting enhanced communication activity of stromal cells. (**C**) Heatmap (or dot plot) showing the relative strength of interactions between stromal cells and other uterine cell populations, including endothelial cells, and smooth muscle cells. (**D**) Inferred LAMININ signaling intercellular communication network between mock group and PEDV-infected group shown in a circular plot (left). Thicker lines show a higher number of interactions and greater interaction strength between cell types. Expression levels of LAMININ pathway genes in each cell type are compared between control (orange) groups and PEDV-infected (cyan). Normalized expression levels are determined in a violin plot (right). (**E**) Inferred FN1 signaling intercellular communication network between mock group and PEDV-infected group shown in a circular plot (left). Normalized expression levels of FN1 pathway genes are determined in a violin plot (right). (**F**) Inferred COLLAGEN signaling intercellular communication network between mock group and PEDV-infected group shown in a circular plot (left). Normalized expression levels of COLLAGEN pathway genes are determined in a violin plot (right).

To further elucidate how PEDV infection reshapes the uterine cellular microenvironment, we performed ligand–receptor-based cell–cell interaction analysis using single-cell transcriptomic data. Global interaction profiling revealed extensive intercellular communication among major uterine cell populations, with uterine stromal cells emerging as a central signaling hub (Fig. 4A and B). Comparative analysis demonstrated that stromal cells exhibited markedly decreased outgoing and incoming signaling interactions following PEDV infection (Fig. 4A through C). In particular, stromal cells showed predicted communication with perivascular cells with high ISG expression and other perivascular cells (Fig. 4A and C). At the pathway level, stromal cell-mediated interactions were enriched for signaling axes associated with inflammatory responses, antiviral defense, and tissue remodeling, including LAMININ, FN1, and COLLAGEN signaling pathway (Fig. 4D through F). Notably, multiple chemokine- and cytokine-related ligand–receptor pairs were also identified between stromal cells and the residual cells in the culture system, suggesting a potential role for infected stromal cells in shaping the endometrium (S3 Fig). Concurrently, interactions between stromal cells and endothelial cells were dominated by pathways implicated in vascular activation and permeability, potentially facilitating cell trafficking (Fig. 4D through F). In contrast, uterine epithelial cells displayed relatively limited interaction strength with other cell populations, consistent with their minimal susceptibility to PEDV infection observed in the viral transcript analysis (Fig. 4D through F). Together, these findings indicate that PEDV infection selectively activates uterine stromal cells, which function as key signaling orchestrators decreasing intercellular communication.

### PEDV infection significantly alters multiple cells signaling in PPEC

Consequently, we performed RNA sequencing on more samples to further validate the reliability of the above results. Extensive RNA-seq analysis of PPEC showed that PEDV infection caused significant changes in gene expression. Compared with the control group, the expression of 2,508 genes was upregulated, and the expression of 2,361 genes was downregulated in PPEC infected with PEDV CV777 (Fig. S4A). Compared with the mock group, the expression of 2,046 genes was upregulated and the expression of 1,896 genes was downregulated in PPEC infected with PEDV GH (Fig. S4B).

**Fig 5:**
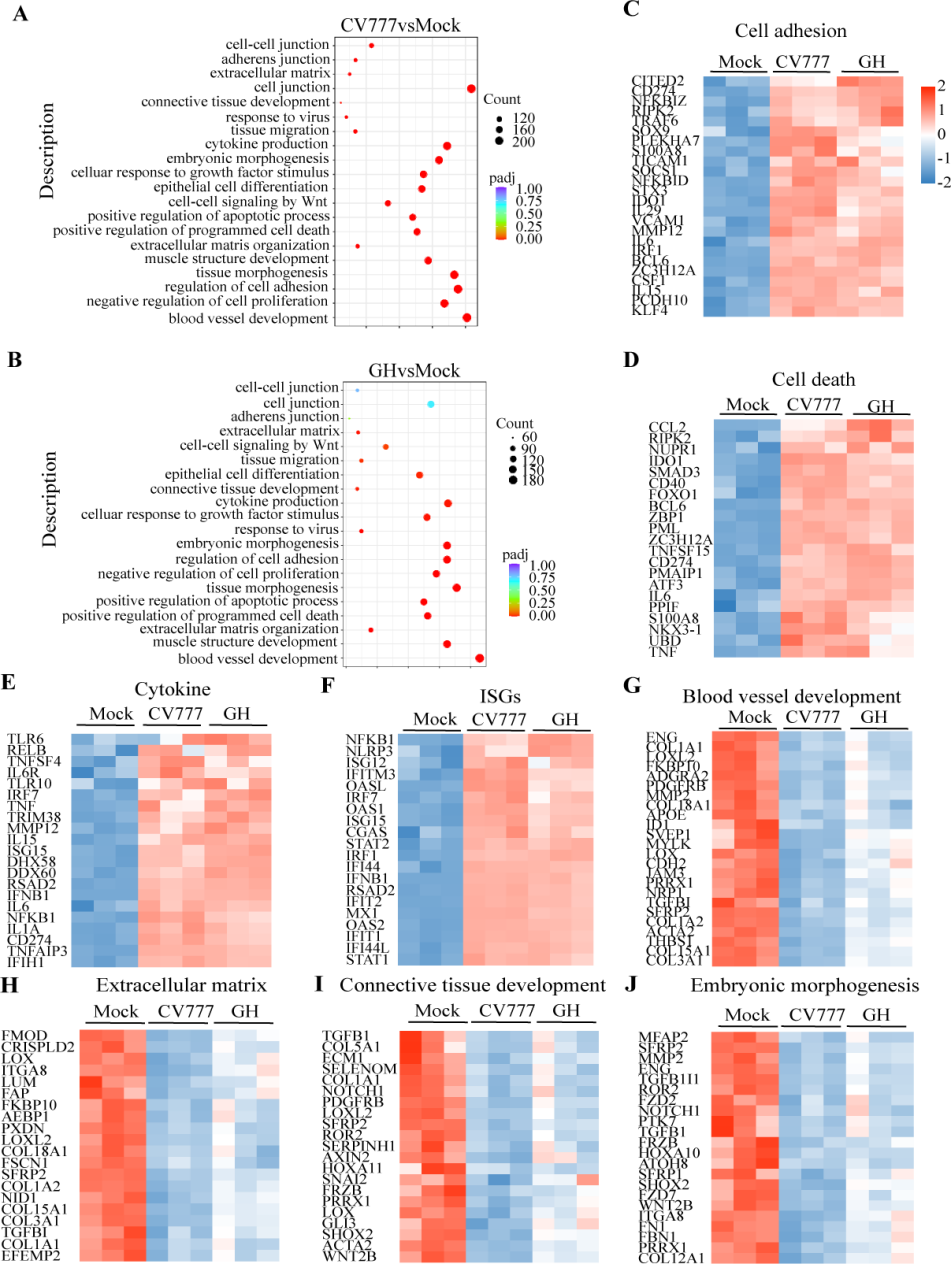
PEDV infection significantly alters multiple cells signaling in PPEC. The bubble size indicates the absolute gene counts enriched in a GO term, and the color bar indicates the minus logarithm of *P* values (**A and B**). C–J Heatmaps indicate the relative expression level for sets of cell adhesion (**C**), cell death (**D**), cytokines (**E**), ISGs (**F**), blood vessel development (**G**), extracellular matrix (**H**), connective tissue development (**I**), and embryonic morphogenesis (**J**).

Gene ontology (GO) functional enrichment analysis of differentially expressed genes (DEGs) showed that the gene sets regulating cytokine signaling transduction and production, the activation of cell adhesion, inflammatory response, innate immunity, and cell death were significantly upregulated. On the contrary, the gene sets that are involved in blood vessel development, extracellular matrix, connective tissue development, and embryonic morphogenesis were downregulated (Fig. 5A and B). We cataloged core differentially expressed genes (DEGs) with significant differences in expression. PEDV infection of PPEC resulted in the downregulation of a large number of genes encoding regulators of cell adhesion (Fig. 5C). Meanwhile, core DEGs associated with cell death showed a trend of upregulation (Fig. 5D). In addition, PEDV infection with PPEC resulted in the upregulation of the expression of antiviral immune genes, especially the significant upregulation of ISGs and IFN-I response genes (Fig. 5E). Besides, the expression of chemokines and inflammatory cytokines was increased (Fig. 5F). A large number of genes encoding regulators of blood vessel development were downregulated after PEDV infection of PPEC (Fig. 5G). Multiple core DEGs associated with the extracellular matrix also showed a trend of downregulation (Fig. 5H). In addition, core DEGs associated with connective tissue development were downregulated, including genes encoding stromal cell proteins (e.g., Col1a1, HOXA11, and Col1a1) (Fig. 5I). Notably, embryonic morphogenetic processes were also downregulated (Fig. 5J).

In summary, the transcriptome data showed that PEDV infection significantly changed a variety of cellular signaling in PPEC, especially upregulating cell adhesion, cell death, immune responses, and inflammation and downregulating cellular signaling associated with blood vessel development, extracellular matrix, connective tissue development, and embryonic morphogenesis, which are crucial in maintaining a normal pregnancy. These results all point to a close association between PEDV infection and reproductive disorders.

### PEDV infection upregulates pro-inflammatory cytokine expression and cellular homeostasis disruption in PPEC

**Fig 6:**
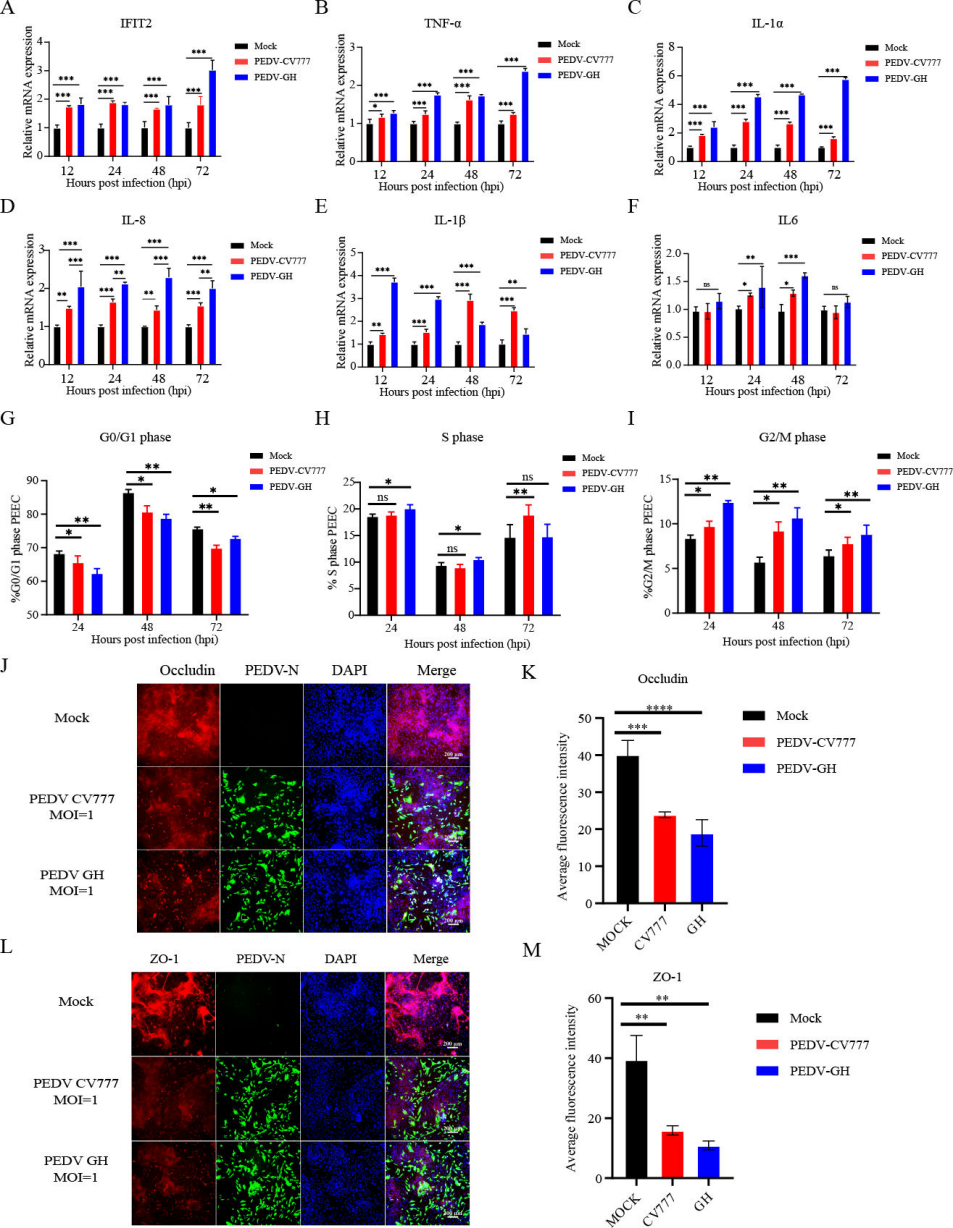
PEDV infection induces immune activation and cellular homeostasis disruption in PPEC. Chemokine expression in PPEC infected with PEDV: PEDV infection enhanced the mRNA expression of IFIT2 (**A**), TNF-α (**B**), IL-1α (**C**), IL8 (**D**), IL-1β (**E**), and IL-6 (**F**) in PPEC at 12, 24, 48, and 72 hpi analyzed by RT-PCR. Effects of PEDV infection on cell cycle regulation of PPEC: the design of the flow cytometry experiment involved three samples per group at 24, 48, and 72 hpi (**A**). The number of PPEC in the G0/G1 phase was significantly less than that of PPEC infected with PEDV CV777 or PEDV GH at 24, 48, and 72 hpi (**G**). The number of PPEC infected with PEDV in the S phase at 24, 48, and 72 hpi (**H**). The number of PPEC infected with PEDV in the G2 phase at 24, 48, and 72 hpi (**I**). PEDV infection disrupts the epithelial tight junctions of PPEC: The levels of occludin in PPEC infected with PEDV were assessed by IFA (**J**). The average fluorescence intensity of occludin in PPEC infected with PEDV was analyzed (**K**). The levels of ZO-1 in PPEC infected with PEDV were assessed by IFA (**L**). The average fluorescence intensity of ZO-1 in PPEC infected with PEDV was analyzed (**M**).

In order to further verify the changes in chemokine expression in PPEC at 12, 24, 48, and 72 h after PEDV infection, we examined the mRNA levels of chemokines in PPEC. Compared with the control group, the PEDV CV777 or PEDV GH-infected cells had significantly induced mRNA expression of IL-1α, IL-1β, IL-6, IL-8, IFIT2, and TNFα (Fig. 6A through F). Such enhancement demonstrated that PPEC can upregulate the expression of pro-inflammatory cytokines after PEDV infection, resulting in a strong immune response to PEDV infection.

The design of the flow cytometry experiment in PPEC infected with PEDV involved three samples per group at 24, 48, and 72 hpi. At 24, 48, and 72 hpi, the number of PPEC infected with PEDV CV777 or PEDV GH in the G0/G1 phase was significantly less than that of normal PPEC (Fig. 6G). Compared to the PPEC, the number of PPEC infected with PEDV CV777 or PEDV GH in the G0/G1 phase was slightly increased (Fig. 6H). Besides, the number of the PPEC infected with PEDV CV777 or PEDV GH in the G2/M phase was significantly increased than that of normal PPEC (Fig. 6I). These results suggest that PPEC infected with PEDV affect the normal cell cycle regulation to some extent, and it contributes to G0 and G2 phase abnormality.

The permeability and integrity of the uterine endometrial barrier are mainly controlled by tight junctions (13). Thus, the expression of uterine endothelial tight junctions was determined after incubation of PPEC with PEDV CV777 and GH at 48 hpi. In comparison with the mock group, overall, PPEC infected the PEDV CV777 and PEDV GH reduced the expression of zonula occludens-1 (ZO-1) and occludin (Fig. 6J and L). We analyzed the average fluorescence intensity and found that the fluorescence intensity of both occludin and ZO-1 was significantly reduced in PPEC infected with PEDV CV777 and PEDV GH compared to the mock group (Fig. 6K and H). From a local perspective, the lack of tight junction proteins around viral particles could be primarily due to the disruption of tight junctions caused by viral infection (Fig. 6J and L). These results indicate that PEDV exerts a destructive effect on the uterine endothelial barrier through a direct role of the virus.

### Correlation analysis between PEDV gene expression and known coronavirus receptor expression across uterine cell populations

**Fig 7:**
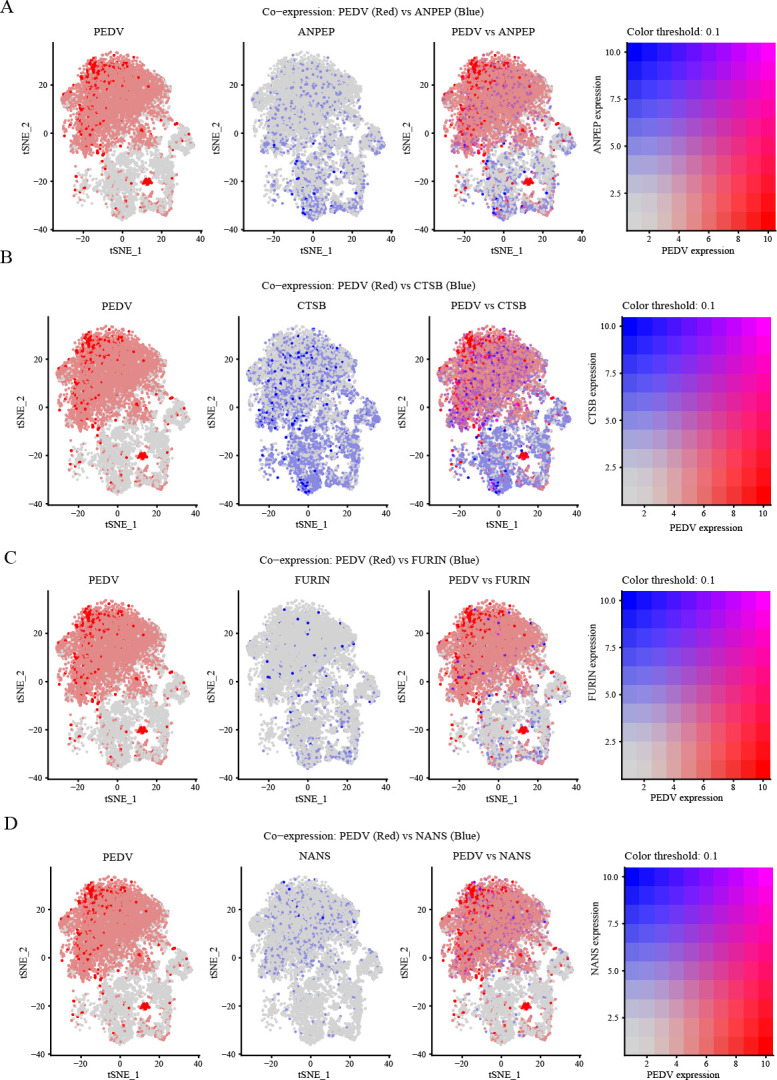
Correlation analysis between PEDV gene expression and known coronavirus receptor expression across uterine cell populations. Single-cell RNA sequencing data from PEDV-infected porcine uteri were used to assess the relationship between viral gene expression and host receptor genes associated with coronavirus entry. The UMAP visualization showing the expression levels of ANPEP (**A**), CTSB (**B**), FURIN (**C**), and NANS (**D**) in control and PEDV-infected groups. Each dot represents an individual cell, colored by cell type. Correlation coefficients were calculated across all cells to evaluate the association between PEDV expression levels and receptor gene expression.

Single-cell RNA sequencing revealed that PEDV infection predominantly occurred in uterine stromal cells. To further explore potential host factors associated with PEDV tropism, we performed a correlation analysis between PEDV gene expression and the expression of known coronavirus entry-associated receptors across all identified uterine cell populations. The analysis demonstrated that PEDV expression showed a weak positive association with ANPEP, CTSB, and NANS enriched in uterine stromal cells, whereas epithelial, endothelial, and stem cell populations exhibited negligible correlations (Fig. 7A, B and D). Notably, although the overall correlation coefficients were 0.1 (Fig. 7 and Fig. S5), NANS displayed consistent positive trends specifically within PEDV-positive stromal cells (Fig. 7D), suggesting cell-type-restricted co-expression patterns rather than global transcriptional coupling.

Although the correlation coefficients were relatively low, such magnitudes are commonly observed in single-cell data sets due to transcriptional sparsity and cellular heterogeneity and nevertheless may reflect biologically meaningful cell-type-specific associations. A Pearson correlation of 0.1 indicates a very weak or negligible positive linear relationship between PEDV viral transcript abundance and the expression of these host genes across the analyzed uterine cell populations. This threshold (0.1) is used as the color threshold in the co-expression panels, suggesting minimal spatial overlap in their expression patterns. Collectively, these results indicate that PEDV infection in the uterus is closely associated with the transcriptional landscape of stromal cells and that receptor expression patterns may contribute to the observed cellular tropism.

## DISCUSSION

In this study, we identify uterine stromal cells as a previously unrecognized cellular target of PEDV infection *in vivo* and *in vitro*. While PEDV has long been considered a strictly enteric virus (6, 7), our data reveal that viral infection extends beyond the intestinal tract and involves the uterine microenvironment. Through an integrated approach combining single-cell transcriptomics, primary cell infection models, and *in vivo* validation, we demonstrate that PEDV exhibits a marked preference for stromal cells within the uterus. These findings expand the tissue and cellular tropism of PEDV and highlight an extraintestinal facet of alphacoronavirus pathogenesis.

**Fig 8:**
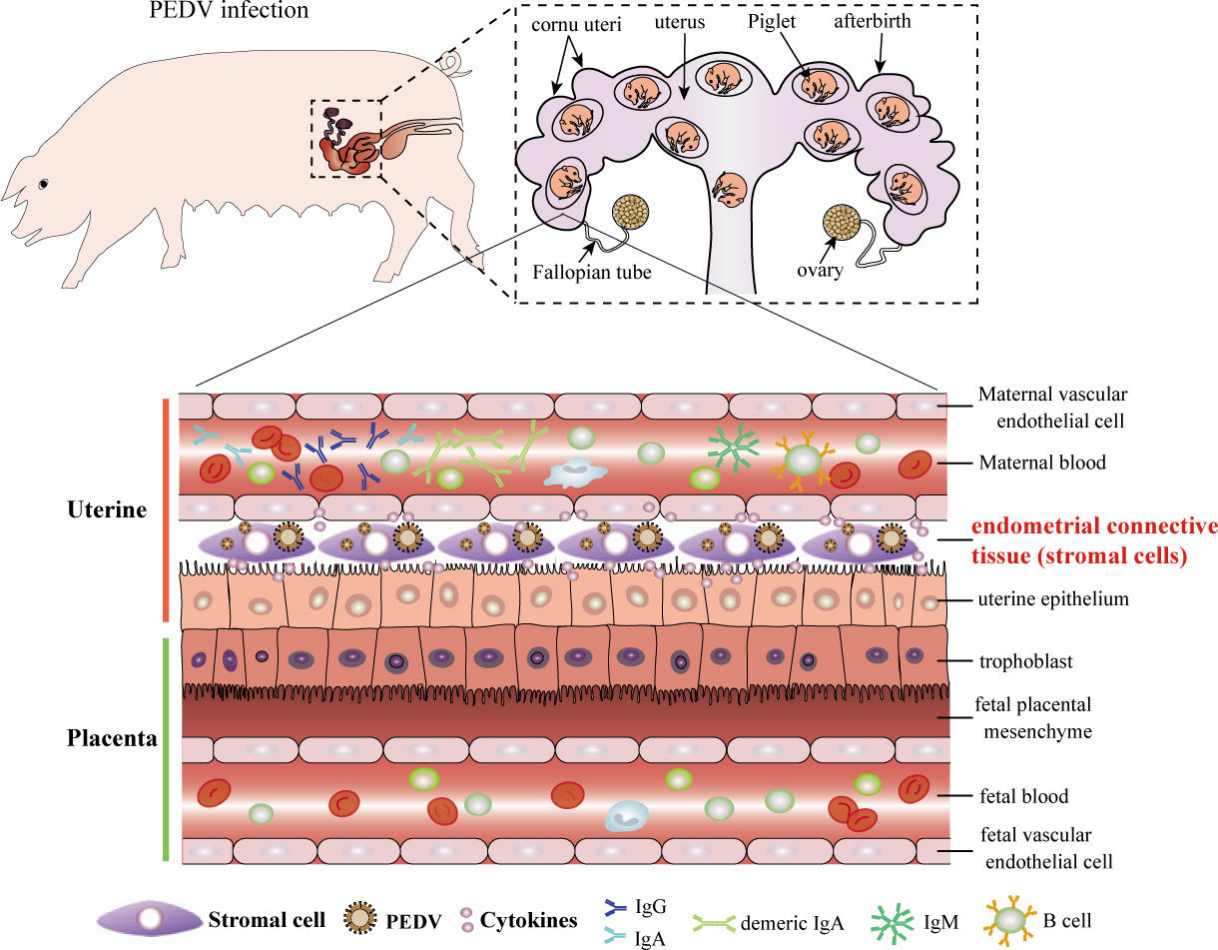
Overview of pregnant sow infected with porcine epidemic diarrhea virus. The placental barrier of sows in the late gestation period is mainly composed of these six types of tightly linked cells (maternal vascular endothelial cell, stromal cell, uterine epithelium, trophoblast, fetal placental mesenchyme, and fetal vascular endothelial cell). PEDV can successfully infect stromal cells and replicate efficiently, which may contribute to reproductive disorders through induction of inflammatory responses, extracellular matrix disruption, and altered intercellular communication.

Whether PEDV can cross the placental barrier remains to be verified; however, the transcriptional and functional alterations observed in PEDV-infected uterine stromal cells provide a plausible mechanistic link to the reproductive disorders documented in field outbreaks (Fig. 8). The clinical symptoms seen in infected sows, including embryonic death, weak-born piglets, late-term abortions, and early farrowing, may, therefore, be partially attributed to uterine stromal cell infection (Fig. 8). Furthermore, cell–cell interaction analysis suggests that PEDV infection reprograms uterine stromal cells into active signaling hubs that function as key signaling orchestrators, decreasing intercellular communication.

Although a direct causal link between stromal cell infection and adverse pregnancy outcomes cannot be conclusively established in the present study, in part because establishing a pregnant sow challenge model with targeted uterine infection remains technically challenging, several lines of evidence support a functional association. Uterine stromal cells play essential roles in maintaining endometrial integrity, supporting implantation, and orchestrating immune–vascular interactions during pregnancy (14, 23). PEDV infection of these cells is accompanied by pronounced transcriptional reprogramming, including inflammatory activation, disruption of extracellular matrix-related pathways, and altered intercellular communication networks. Collectively, these changes are consistent with a uterine environment that may be unfavorable for normal reproductive processes. Our cell–cell communication analysis further suggests that PEDV infection reprograms uterine stromal cells into active signaling hubs. These analyses reveal potential signaling interactions between stromal cells and vascular cell populations, particularly through pathways linked to inflammation, matrix remodeling, and tissue organization. It is important to note that these analyses were performed on primary cultured endometrial cells rather than on freshly dissociated intact sows uterine tissue infected with PEDV. Immune cell populations were largely lost during the adherent culture, washing, and passaging processes, which precluded direct assessment of immune cell recruitment, activation status, or spatial interactions with infected stromal cells *in vivo*. Consequently, although we observed robust intrinsic inflammatory activation in PEDV-infected stromal cells and predicted potential stromal–immune signaling axes, these findings require further validation using freshly isolated uterine tissue samples from PEDV-infected pregnant sows.

A notable finding of this study is that PEDV efficiently infects and replicates in primary uterine endometrial cells in the absence of exogenous trypsin. This contrasts with the established requirement for protease activity during intestinal infection (24, 25) and suggests that PEDV entry mechanisms may be tissue specific. The uterine microenvironment likely provides alternative host proteases or endosomal conditions that facilitate viral entry and fusion (26–28). These observations underscore the adaptability of PEDV to distinct tissue contexts and warrant further investigation into non-canonical entry pathways in extraintestinal sites.

We further explored the relationship between PEDV infection and the expression of known coronavirus entry-associated genes at single-cell resolution (24, 25, 28–30). Although correlation coefficients were modest, consistent with the sparsity and heterogeneity of single-cell data sets, PEDV expression showed preferential associations with stromal cell-enriched host factors rather than canonical epithelial coronavirus receptors. These findings do not identify a definitive entry receptor but instead suggest that PEDV infection in the uterus occurs within a distinct cellular and molecular context. Future functional studies will be required to determine whether these candidate host factors contribute directly to viral entry or replication.

A limitation of our study is that we did not directly investigate the causal relationship between PEDV infection of uterine stromal cells and adverse reproductive outcomes in a controlled *in vivo* pregnancy model. We acknowledge that establishing and maintaining a pregnant sow challenge model is technically demanding, which limited our ability to perform such experiments in the present study. Nevertheless, our single-cell RNA sequencing analysis of isolated mixed uterine cells provides a relatively comprehensive characterization of the extra-intestinal infection features of PEDV and reveals the susceptibility of uterine stromal cells to viral infection. Although our findings suggest a potential link between uterine infection and reproductive disorders, definitive evidence of causality remains to be established. Future studies employing pregnant sow models with targeted uterine infection will be necessary to determine whether PEDV infection of uterine stromal cells is sufficient to induce reproductive dysfunction. Such investigations will facilitate a deeper understanding of the extra-intestinal pathogenic mechanisms of PEDV. And these results also provide a conceptual framework for understanding PEDV-associated reproductive disorders and highlight uterine stromal cells as a critical, yet previously overlooked, component of alphacoronavirus pathology.

## MATERIALS AND METHODS

### Animals

To detect whether there is PEDV in the uterus, we collected three pregnant sows that were naturally infected with PEDV from a pig farm experiencing a PEDV outbreak. These sows infected with PEDV had given birth to piglets for 1 week, and the piglets suffered from severe diarrhea and were positive for PEDV. To isolate primary porcine uterine endometrial cells, the nine adult sows (control group) of the specific-pathogen free (SPF) from three different farms were used in this study. The nine adult pigs of the specific-pathogen free (SPF) grade were monitored daily for any changes in behavior or health status. Immediately after the five intact uterine organs were harvested, they were immersed in the PBS supplemented with antibiotics (streptomycin 100 mg/mL, penicillin 100 U/mL, amphotericin B 5 mg/mL).

### Isolation of primary porcine endometrial cells

Each uterine organ was transferred into the petri dish, exposed, and opened longitudinally with a big pair of scissors. Subsequently, the upper layer of the endometrium is cut off with a small pair of scissors and transferred to a new petri dish. Then, the resulting epithelium of the endometrium was cut into 1 cubic millimeter pieces on a new petri dish. The tissue pieces were washed 1–2 times with PBS and then centrifuged to discard the supernatant. Add twice the volume of 0.1 mg/mL of collagenase type 1 into the tissue and incubate at 37°C for 2.5 h, during which time take out and shake several times every 30 min. The digested cells were added to DMEM medium (DMEM, Gibco-BRL) containing 10% fetal bovine serum (Excell, China) in equal volume to terminate digestion, and then the cell samples were passed through 100 mesh cell screens to obtain primary endometrial cells. A portion of the primary endometrial cells obtained was frozen with cell freeze solution for future experimental use.

### Cell culture

DMEM/F12 -Dulbecco’s modified Eagle medium (Gibco) containing 20% fetal bovine serum (FBS, SORFA Life Science), 2% penicillin-streptomycin (Gibco), and Insulin (1 U/mL) was used for maintaining the primary endometrial cells, which were incubated at 37°C in 5% CO_2_. The insulin in this paper was replaced by human insulin (Yisheng, China). The porcine intestinal epithelial cell line J2 (IPEC-J2) cells and African green monkey epithelial (Vero) cells were collected in our laboratory. The Vero cells and IPEC-J2 cells were cultured in DMEM medium (Gibco) supplemented with 10% FBS. All cells were checked for mycoplasma contamination using Kit (Yisheng, China).

### Single-cell RNA sequencing (scRNA-seq)

Two samples (the primary porcine uterine endometrial cells [PPEC] and PPEC infected with PEDV) were collected and promptly processed to obtain viable single-cell suspensions. At 48 h post-infection (hpi) with the PEDV GH strain at a multiplicity of infection (MOI) of 1, the infected cells were mechanically dissociated and subsequently subjected to enzymatic digestion in a buffer containing collagenase and DNase I. After digestion, the cell suspensions were filtered through a 40-μm mesh to remove debris and aggregates. Cell viability was examined using trypan blue exclusion, and only suspensions with over 85% viable cells were subjected to sequencing.

Single-cell transcriptomic libraries were constructed using the Chromium platform (10× Genomics, USA) following the manufacturer’s workflow, with cell encapsulation, barcoding, and cDNA synthesis carried out within individual Gel Beads in Emulsion (GEMs). The resulting libraries were amplified, purified, and quantified prior to sequencing on an Illumina NovaSeq 6000 instrument with paired-end reads.

Raw sequencing files were processed with the Cell Ranger software suite (10× Genomics) to demultiplex cell barcodes, align reads to the reference genome (*sus_scrofa__ensembl-108_Sscrofa11.1*), and generate digital gene expression matrices. Subsequent quality control and bioinformatic analyses, including normalization, clustering, dimensionality reduction (PCA and UMAP), and differential expression testing, were performed in R using the Seurat package. Initially, stringent quality control (QC) metrics were applied, with the mitochondrial gene percentage capped at 5%, effectively excluding low-quality droplets derived from cell lysis—the primary source of ambient RNA. Cells with fewer than 200 detected genes or with mitochondrial transcript content greater than 20% were removed from the data set to ensure data reliability.

### Cell–cell ligand–receptor interaction analysis

The integrated Seurat object was used for cell–cell communication analysis, with annotated cell types set as identities. Cells were divided into mock and PEDV groups according to sample information and analyzed separately. Because the CellChat database is based on human ligand–receptor pairs, porcine genes were first mapped to human homologs using BLASTP, and only mappings with amino acid identity >30% were retained. For each group, normalized gene expression data were extracted from the RNA assay, gene symbols were converted to uppercase, and metadata containing cell-type and group information were generated. CellChat objects were then constructed using the human ligand–receptor database (CellChatDB.human). Intercellular communication probabilities were inferred at the ligand–receptor level (raw.use = TRUE, population.size = TRUE), with interactions detected in fewer than 10 cells removed. Communication probabilities were further summarized at the signaling pathway level to generate global cell–cell communication networks. Network centrality analysis was performed to identify key signaling cell populations, and the resulting networks were compared between the mock and PEDV groups.

### PEDV propagation and titration

In 2018, the PEDV GH strain (MG983755), belonging to G2 subtype, was isolated in our laboratory. The PEDV CV777 strain (AF353511.1), belonging to G1 subtype, was isolated from an attenuated vaccine in our laboratory. The PEDV GH and PEDV CV777 were propagated by infecting Vero cells at a multiplicity of infection (MOI) of 0.1. Cell cytopathic effects (CPE) caused by PEDV infection were observed at 48 and 72 h post infection (hpi). When Vero cells infected with PEDV have 70%–80% CPE, the cell was frozen at −80℃, and viral titration was measured using tissue culture infective dose 50 (TCID_50_) (31).

### Antibodies

The following antibodies were used in this study: mouse anti-PEDV N protein monoclonal antibody (M100048, Zoonogen, Beijing, China), FITC-488-conjugated Affinipure goat anti-mouse IgG (H + L) secondary antibody (SA00013-1; Proteintech, USA), occludin antibody (502601, ZEN BIO), ZO-1 antibody (40-2200, Thermo Fisher scientific), Anti-wide spectrum Cytokeratin (ab9377, abcam), CD10 Rabbit Monoclonal Antibody (AC0010, Beyotime, China), CD10 Rabbit Monoclonal Antibody (18008-1-AP, Proteintech, USA), CD10 Rabbit Monoclonal Antibody (GB114689, Servicebio, China), and Vimentin Rabbit Monoclonal Antibody (AF1975, Beyotime, China).

### Immunofluorescence assay

The primary porcine uterine endometrial cells were inoculated in 24-well plates or 48-well plates. When the cells grew to 80% confluency, they were infected with PEDV at MOI = 1 or MOI = 0.1. At 48 hpi, cells were fixed with 4% paraformaldehyde at 4°C for 30 min. Then, 0.5% Triton X-100 was used to penetrate the cells for 10 min at 37℃. Subsequently, the cells were blocked with 1% Bovine Serum Albumin (BSA) at 37°C for 1 h. After that, the mouse anti-PEDV-N protein monoclonal antibody (1:200 dilution) was incubated with the cells at 37°C for 1 h, and then cells were incubated with the FITC 488 goat anti-mouse IgG antibody (1:200 dilution) at 37°C for 1 h. The cell nuclei were then stained by DAPI (Beyotime, China). Finally, the cells were observed using a fluorescence microscope.

### Quantitative real-time reverse transcription-PCR

Total RNA was extracted from the primary endometrial cells infected with PEDV using a Total RNA extracting Plus Kit (Magen, China). The synthesis of the first-strand cDNA was obtained using the GoScript Reverse Transcription Mix kit (Promega, China). The transcription levels of the target genes were determined by qRT-PCR using Eastep qPCR Master Mix kit (Vazyme, China). The PEDV titer is converted into the virus copy number based on the standard curve made by the positive plasmid (PCMV-PEDV-N plsmid). The primers used for qRT-PCR are given in Table S1. Relative expression levels of the target genes were determined using the 2^−ΔΔ^Ct threshold method.

### Western blotting

The primary porcine uterine endometrial cells were grown in a 6-well plate. When the cells infected with PEDV CV777 and PEDV GH at 48 hpi or 72 hpi, then the cells are lysed and the protein were obtained. The rest of the experimental procedure is the same as the common Western blotting test procedure, and the rest of the experimental was also described previously (32).

### RNA sequencing and data analysis

The primary porcine uterine endometrial cells (PPEC) were infected with PEDV CV777 and PEDV GH at 48 hpi in MOI = 0.5. Total RNAs of PPEC were extracted using Trizol. Differentially expressed genes (DEGs) between the PEDV and mock groups were identified using the *FindMarkers* function. Genes with *P* < 0.05 and |log₂ fold change| > 0.5 were considered significantly differentially expressed. Gene Ontology (GO) enrichment analysis was subsequently performed on the DEG sets using the *clusterProfiler* package. The rest of the experiment was completed in the Novogene company.

### Statistical analysis

Differences between the experimental groups and the control group were analyzed by two-way analysis of variance (ANOVA) using the least-square difference multiple-comparison test, which was analyzed statistically significant at *P* < 0.05 and *P* < 0.01. The data were presented using the mean ± the standard deviation (SD) and GraphPad 9.0 software.

## Data Availability

The raw data of RNA sequencing reported in this paper have been deposited in the Genome Sequence Archive (Genomics, Proteomics & Bioinformatics 2025) at the National Genomics Data Center, China National Center for Bioinformation/Beijing Institute of Genomics, Chinese Academy of Sciences (Nucleic Acids Res 2025), under accession number CRA019610. The scRNA-seq data generated in this study have been deposited in the Genome Sequence Archive of the National Genomics Data Center (NGDC) under accession number CRA034218. The data are publicly accessible at https://ngdc.cncb.ac.cn/gsa.

## SUPPLEMENTAL MATERIAL

Supplemental material jvi.00237-26-s0001.pdf Fig. S1 to S6; Tables S1 to S3.

ASM does not own the copyrights to Supplemental Material that may be linked to, or accessed through, an article. The authors have granted ASM a non-exclusive, world-wide license to publish the Supplemental Material files. Please contact the corresponding author directly for reuse.
