# Supplementary material for: Single-cell RNA sequencing identifies uterine stromal cells as a previously unrecognized target of an alphacoronavirus underlying reproductive disorders
Source: J Virol. 2026 Jul 22;100(8):e00237-26. doi: 10.1128/jvi.00237-26 (PMC13483428; doi:10.1128/jvi.00237-26)
Supplement: Supplementary file 1 [file jvi.00237-26-s0001.pdf]

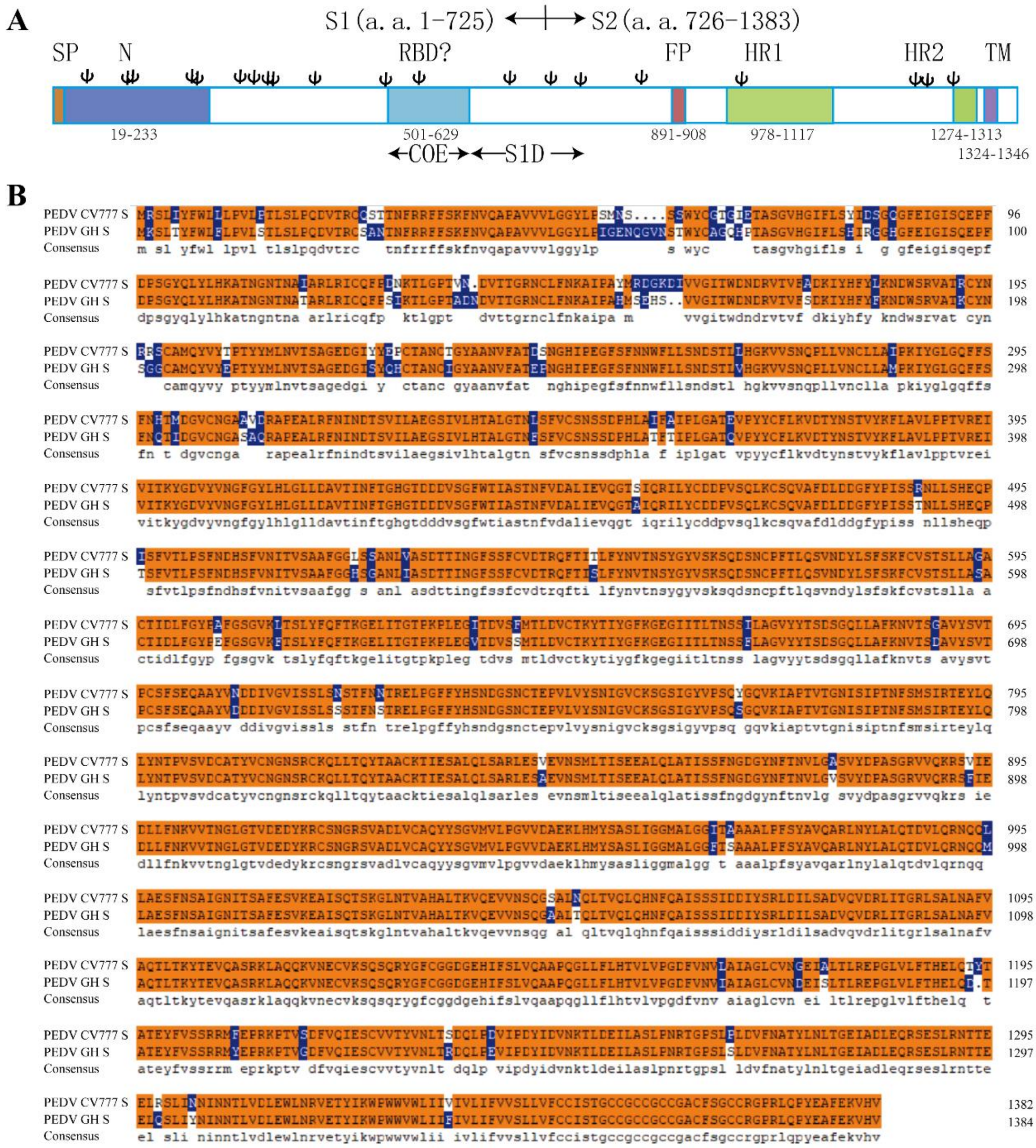

S1 Fig. Schematic presentation of spike proteins of PEDV. (A) The PEDV S protein. The S1 and S2 subunits, the signal peptide (SP, residues 1–18), the N-terminal domain (N, residue 19–233), regions containing neutralizing epitopes (COE, residues 499–638 and S1D, residues 636–789), the fusion peptide (FP; residues 891–908), two heptad repeat regions (HR1, residues 978–1117 and HR2, residues 1274–1313), the transmembrane domain (TM, residues 1324–1346; predicted by TMHMM server) and predicted N-glycosylation sites (predicted by the NetNGlyc server) are indicated with ψ. (B) Analysis of spike proteins of PEDV CV777 and PEDV GH.

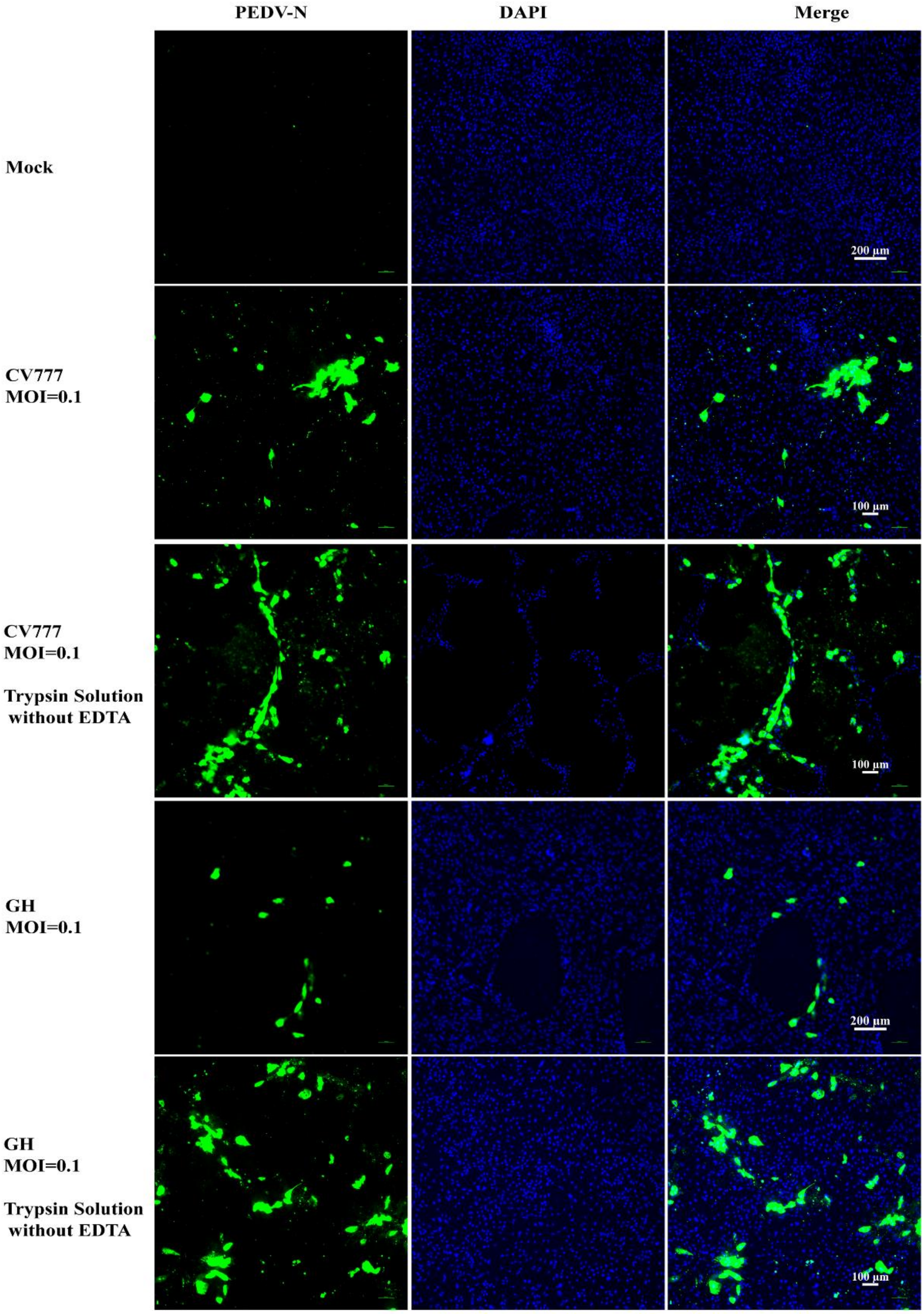

S2 Fig. PEDV efficiently Replicates in PPEC with trypsin solution. IFA indicates PEDV-N protein positive signals in PPEC infected with either CV777 strain or GH strain at MOI=0.1 with trypsin solution was significantly higher than PPEC infected with PEDV without trypsin solution.

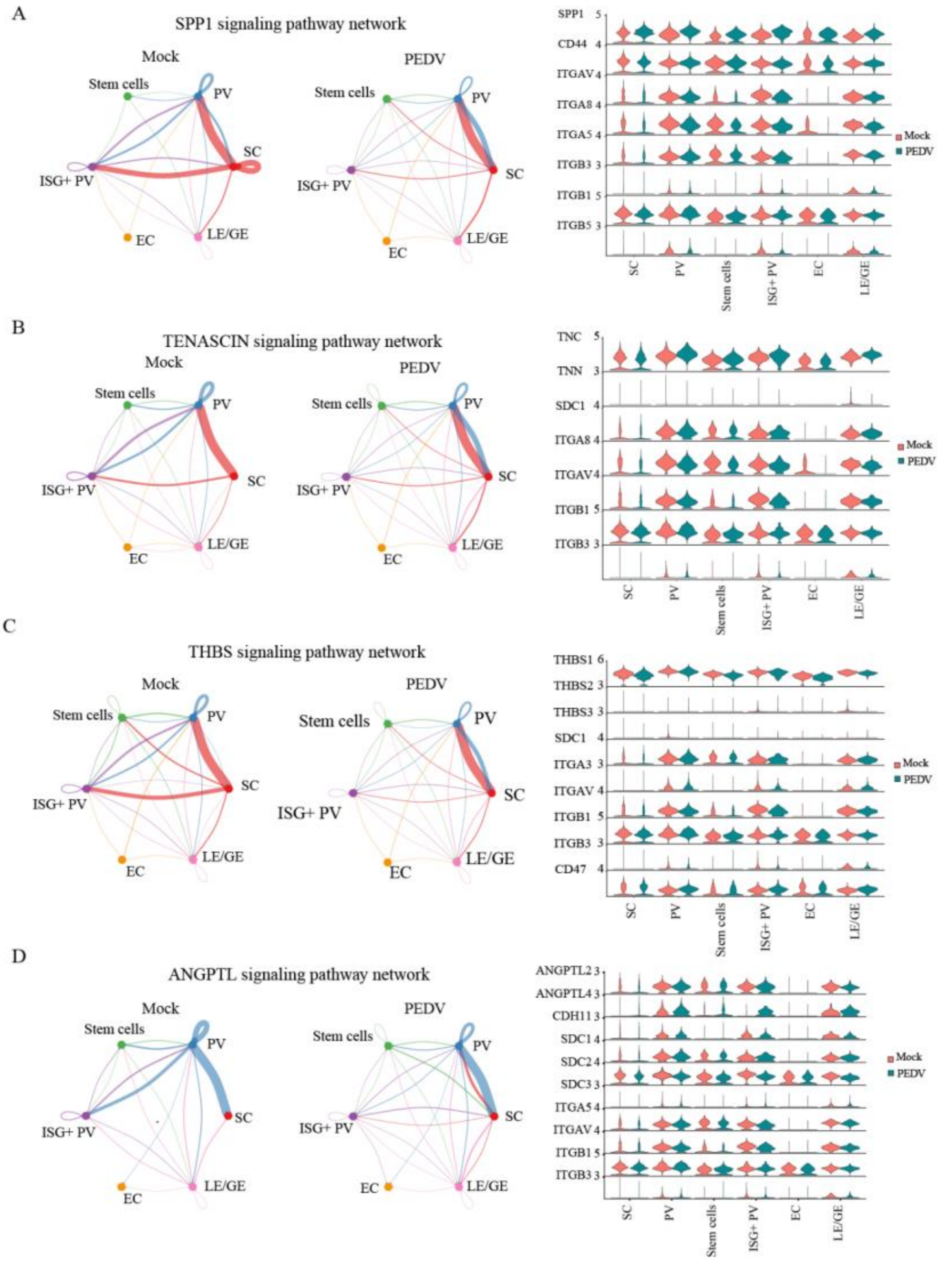

S3 Fig. Cell–cell communication network analysis reveals uterine stromal cells as a central signaling hub following PEDV infection. (A) Inferred SPP1 signaling intercellular communication network between mock group and PEDV infected group shown in a circular plot (left). Thicker lines show a higher number of interactions and greater interaction strength between cell types. Expression levels of SPP1 pathway genes in each cell type are compared between control (orange) groups and PEDV-

infected (cyan). Normalized expression levels are determined in a violin plot (right). (B) Inferred TEANSCIN signaling intercellular communication network between mock group and PEDV infected group shown in a circular plot (left). Normalized expression levels of TEANSCIN pathway genes are determined in a violin plot (right). (C) Inferred THBS signaling intercellular communication network between mock group and PEDV infected group shown in a circular plot (left). Normalized expression levels of THBS pathway genes are determined in a violin plot (right). (D) Inferred ANGPTL signaling intercellular communication network between mock group and PEDV infected group shown in a circular plot (left). Normalized expression levels of ANGPTL pathway genes are determined in a violin plot (right).

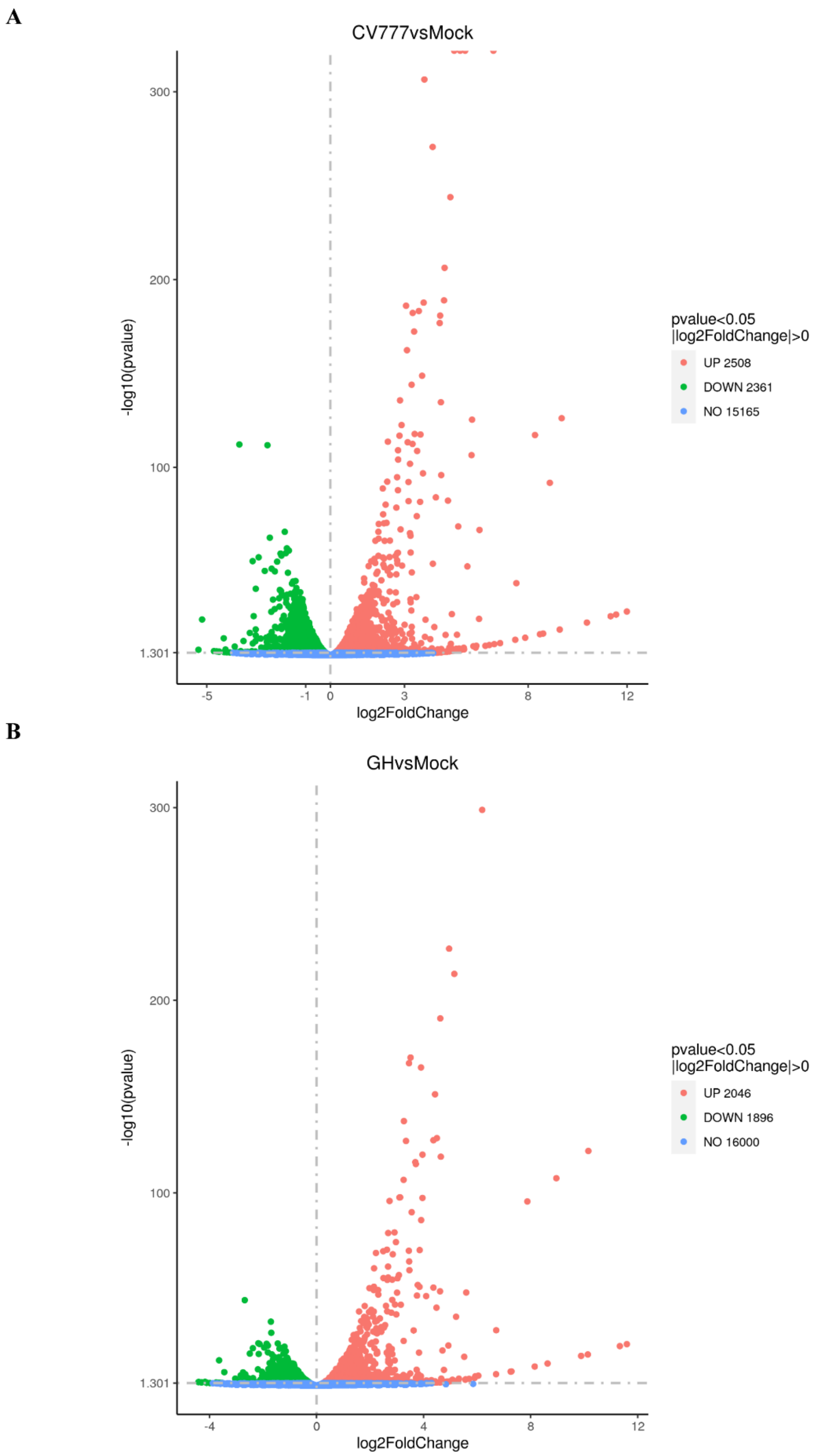

S4 Fig. Transcriptome analysis of PPEC infected with PEDV CV777 and PEDV GH. Volcano plot of DEGs between PPEC infected with PEDV CV777 and Mock (A). Volcano plot of DEGs between PPEC infected with PEDV GH and Mock (B).

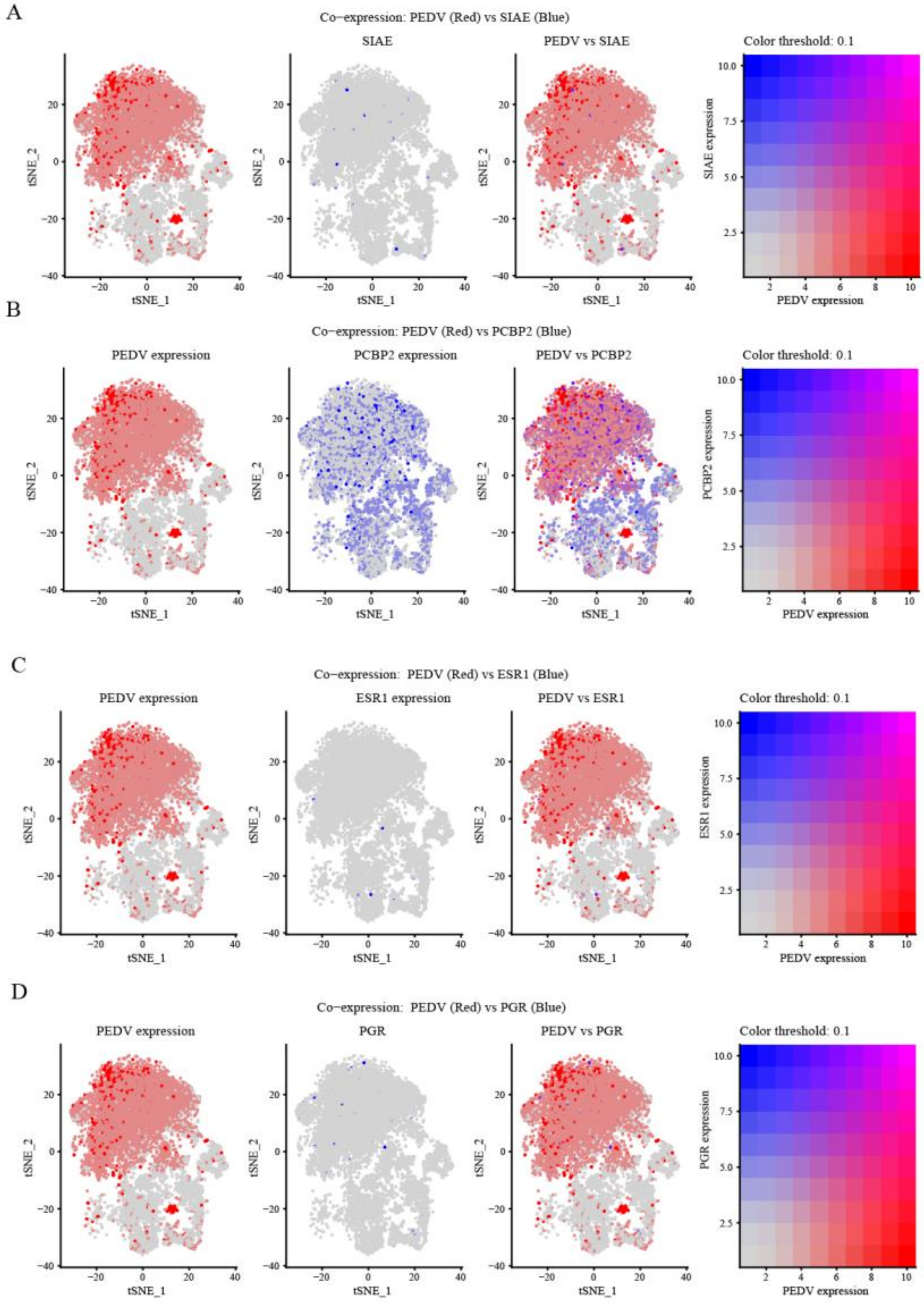

S5 Fig. Correlation analysis between PEDV gene expression and known coronavirus receptor expression across uterine cell populations. Single-cell RNA sequencing data from PEDV-infected porcine uteri were used to assess the relationship

between viral gene expression and host receptor genes associated with coronavirus entry. The UMAP visualization showing the expression levels of SIAE (A), PCBP2 (B), ESR1 (C) and PGR (D) in control and PEDV-infected groups. Each dot represents an individual cell, colored by cell type. Correlation coefficients were calculated across all cells to evaluate the association between PEDV expression levels and receptor gene expression.

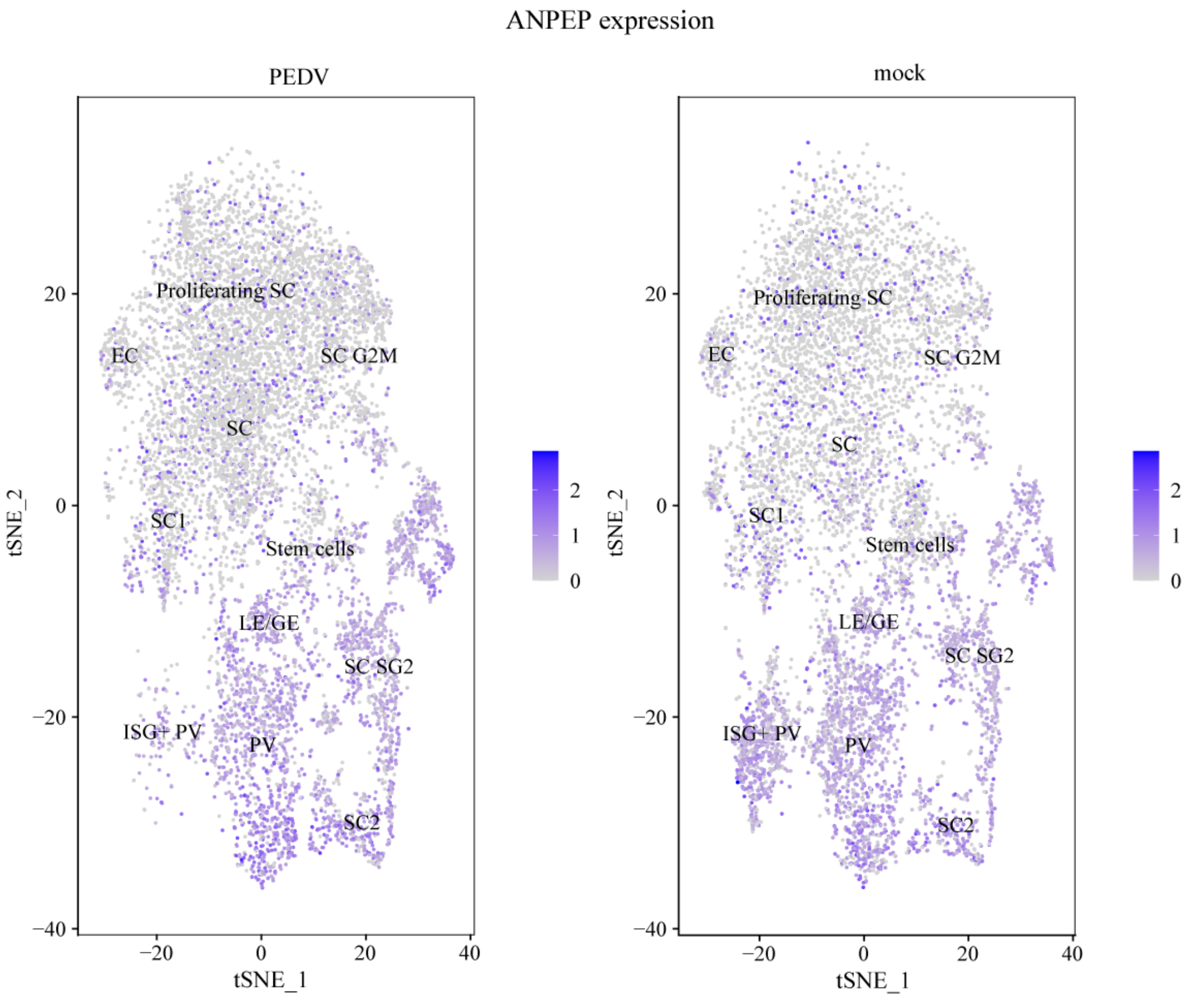

S6 Fig. The UMAP visualization showing the expression levels of ANPEP in control and PEDV-infected groups.

Table S1. Primers used for RT-PCR analysis of pro-infammatory cytokine on PPEC

Gene Names	Prime number	Primer Sequences	Genbank Number
IFIT2	P- IFIT2-F	cctgggaagacttgcagaag	NM_001315658.1
	P-IFIT2-R	ggccagttatccagacggta	
TNF-α	P-TNF-α-F	ccaccaacgttttcctcact	EU682384.1
	P-TNF-α-R	ccaaaatagacctgcccaga	
IL1α	P-IL1α-F	caaggacagtgtggtgatgg	XM_013987857.2
	P- IL1α-R	catcattcaggatgcactgg	
IL8	P- IL8-F	tggcagttttcctgctttct	NM_213867.1
	P- IL8-R	cagtggggtccactctcaat	
IL1β	P-IL1β-F	cagccatggccatagtacct	XM_021085847.1
	P-IL1β-R	ccacgatgacagacaccatc	
IL6	P-IL6-F	caccaggaacgaaagagagc	NM_214399.1
	P-IL6-R	gttttgtccggagaggtgaa	
Actin	P-Actin-F	gccctggactttgaaaatga	EU655628.1
	P-Actin-R	ccccagagaggacgttgtta	

Table S2. The different types of cells in terms of the number and proportion of cells in PPEC in the Mock group

The cells type	The number of cells	Percent
EC	219	2.693%
ISG+ PV	859	10.563%
LE/GE	171	2.103%
PV	1385	17.031%
Stem cells	629	7.735%
Proliferating SC	1528	18.790%
SC G2M	982	12.076%
SC	944	11.608%
SC SG2	504	6.198%
SC1	609	7.489%
SC2	302	3.714%
All SC	4869	59.875%
Total of all cells	8132	100.000%

Table S3. The different types of cells in terms of the number and proportion of cells in PPEC in the PEDV group

The cells type	The number of cells	Percent
EC	264	2.785%
ISG+ PV	132	1.393%
LE/GE	190	2.005%
PV	1123	11.848%
Stem cells	478	5.043%
Proliferating SC	2137	22.547%
SC G2M	1854	19.561%
SC	1555	16.406%
SC SG2	594	6.267%
SC1	790	8.335%
SC2	361	3.809%
All SC	7291	76.926%
Total of all cells	9478	100.000%
